# In Situ Gel Loaded with Chitosan-Coated Simvastatin Nanoparticles: Promising Delivery for Effective Anti-Proliferative Activity against Tongue Carcinoma

**DOI:** 10.3390/md18040201

**Published:** 2020-04-09

**Authors:** Mallesh Kurakula, N. Raghavendra Naveen

**Affiliations:** 1 Department of Biomedical Engineering, The University of Memphis, Memphis, TN 38152, USA; 2 Department of Pharmaceutics, Annamacharya College of Pharmacy, New Boyanapalli, Rajampet, Andhra Pradesh 516126, India; raghavendra.naveen@gmail.com

**Keywords:** simvastatin, chitosan, quercetin, face-centered central composite design, in situ gel, tongue carcinoma

## Abstract

The goal of this study is to develop optimized chitosan-coated Simvastatin (SIM) nanoparticles (NPs) loaded in an in situ gel (ISG) formulation via a face-centered central composite design (FCCCD). Coated SIM-NPs were doped with Quercetin (QRC) using a modified nanoprecipitation method. The concentrations of poloxamer 188 (A) and chitosan (B) at five different levels, plus/minus alpha (+1.414 and −1.414: axial points), plus/minus 1 (factorial points) and the center point were optimized for particle size (PS-Y1), entrapment efficacy (EE-Y2) and stability index (SI-Y3). Based on the desirability approach, a formulation containing poloxamer 188 0.24% and chitosan 0.43% renders the prerequisites of optimum formulation for preparing SIM–QRC NP-loaded ISG. Scanning microscopy showed spherical SIM-NPs, indicating monodispersity in the range of 0.50 ± 0.04 nm with a charge of +32.42 mV. The optimized formulation indicated the highest EE 79.67% and better stability at 4 °C. Drug release from SIM–QRC NP-loaded ISG was slower to plateau by up to 96 h and, at the end of 168 h, only 65.12% of SIM was released in a more controlled manner in comparison to SIM–QRC NPs and plain SIM. ISG formulation showed a considerable increase in apoptosis occurrence through caspase-3 mediation and it also enhanced the tumor suppressor protein levels. Enhanced biological activity of SIM was observed due to QRC enabling promising drug and polymer synergistic interaction. The proposed formulation can provide a breakthrough in localized therapy, overcoming the potential drawbacks of systemic chemotherapy for tongue carcinoma.

## 1. Introduction

Oral cancers are the eighth most common cancers in the world, primarily located in the mouth, tongue or oropharynx [1]. The tongue is the most common site and oral squamous cell carcinoma (OSCC) represents 90%–95% of total intraoral carcinoma malignancies [2]. These arise initially from the lining of the tongue and appear like lumps with red or white spots. Although the exact etiology of tongue cancer is unknown, several risk factors like chewing tobacco, pan, and betel nuts have been identified, but these factors vary in different areas of the world [3,4,5]. The incidence of OSCC is rising in most of central and eastern Europe and the USA and the survival rate is just 30% [6,7,8]. 

The drawbacks and complications in connection with the traditional chemotherapy approach for oral cancer treatment, like the non-specific distribution of chemotherapeutic drugs in the body, ineffective tumor growth inhibition, recurrence and metastasis can be overcome by promising drug delivery systems. Marine extracts such as chitosan have been investigated extensively as drug carriers, owing to its excellent stability, biocompatibility, biodegradability, ease in modification and low toxicity [9]. Nanostructured chitosan can inhibit several tumor establishments and have more retention time in the blood. Chitosan has several reactive amino groups, and this exceptional characteristic can provide a high affinity towards biological site-specific targeting and can also offer interaction with many nanostructured materials to form a huge variety of composites [10]. 

Statins act by inhibiting 3-hydroxy-3-methylglutarylcoenzyme A (HMG-CoA) and are efficient agents in minimizing plasma cholesterol [11,12]. Furthermore, statins can inhibit the growth of tumor cells amplifying intracellular calcium mobilization and the repurposing of statins have been approved in treating many clinical conditions. Simvastatin (SIM) has been identified for its potential in treating several types of cancers by cell cycle arrest, inhibiting tumor metastasis and inducing apoptosis [13,14,15].

QRC, a glycone form of flavonoid glycosides, has been used as a nutritional supplement and has a strong ability to scavenge free radicals due to the presence of two antioxidant pharmacophores in its structure [16,17]. The lipophilic nature of QRC favors crossing cellular membranes and initiating signaling pathways connected with chemoprevention [18]; QRC also induces cell cycle arrest and acts as an autophagy mediator, thus preventing further tumor proliferation. Despite this, QRC has poor water solubility, which limits its application in cancer therapy [19]. Nanoformulation of QRC can be a possible way to enhance its anticancer potential and clinical application. 

Since the invention of nanomedicine, the potential of nanotechnology has been improved in terms of its selectivity and minimization of collateral toxicity to non-malignant cells [20]. Nanoparticles (NPs) coated with chitosan provide a strong interaction with the mucosal surface and promote structural reorganization of the tight junction of epithelial cells, which can increase the mucosal drug transport [21].

In situ gelling systems (ISG) are solutions which, at the time of administration and upon contact with the physiological fluids or mucosa, undergo a sol-to-gel transformation and this change is initiated by the presence of electrolytes and a particular pH or temperature. This mechanism of gelation depends on the type of polymer used and the loaded drug released in a sustained or controlled manner [22]. 

Incorporating SIM NPs into an ISG base can allow controlled drug release to the target tumor site. To our knowledge, SIM, along with QRC, has not been explored before, especially against tongue carcinoma. Consequently, the goal of the present study is to design, develop and characterize an optimized ISG formulation for loaded SIM NPs doped with QRC to obtain sustained drug release and evaluate its effectiveness against human tongue squamous carcinoma cell line (HSC-3) cell lines.

## 2. Result and Discussion

### 2.1. Attenuated Total Reflectance (ATR) Infrared (IR) Spectroscopy 

**Figure 1:**
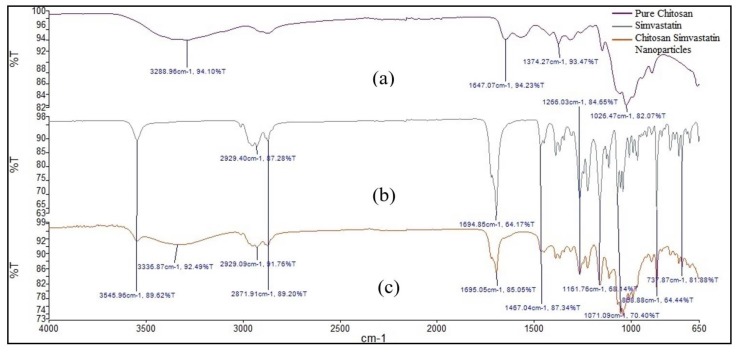
Attenuated Total Reflectance (ATR) Infrared (IR) Spectroscopy (ATR-IR) spectra of (**a**) chitosan (**b**) Simvastatin (SIM) and (**c**) SIM–Quercetin (QRC) nanoparticles (NPs).

Pure SIM, chitosan and SIM–QRC NPs were subjected to Attenuated Total Reflectance (ATR) Infrared (IR) Spectroscopy (ATR-IR) analysis for compatibility studies and the spectra are shown in Figure 1. Functional groups of SIM were found to be unaltered and chitosan bands were also found in the formulation spectra, thus confirming the compatibility between SIM and excipients.

### 2.2. Preparation of Chitosan-Coated SIM–QRC NPs 

The face-centered central composite design (FCCCD) of response surface methodology was applied to assess the optimal levels of the selected variables and their interactions in resulting minimum particle size (PS); maximum entrapment efficacy (EE) and stability index (SI) [23]. A total of 13 runs were projected by the design expert and the observed responses were given in Table 1. The obtained particle size was found to be in the range of 109–354 nm, the calculated EE was about 54.21%–94.51%, while the stability index was estimated to be around 35.45%–92.05%.

**Figure 2:**
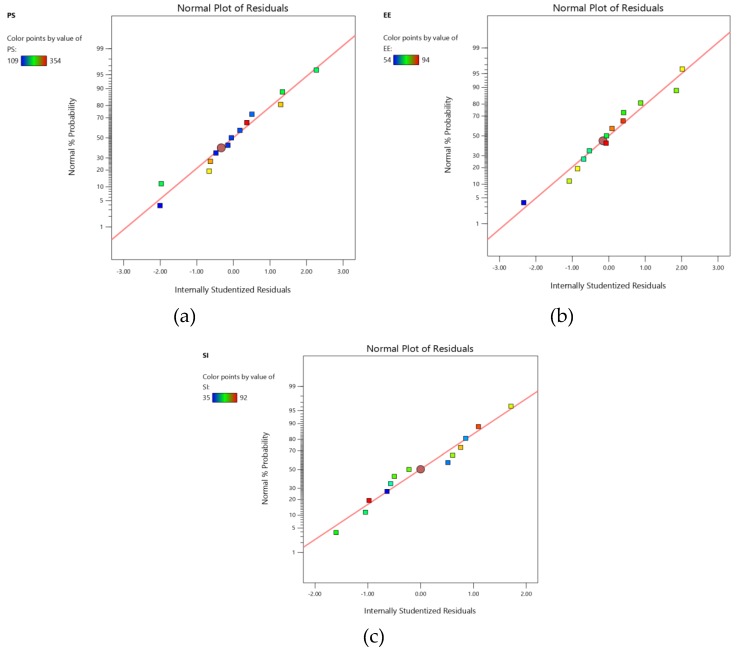
Normal probability plots of the residuals for (**a**) particle size (PS) (**b**) entrapment efficacy (EE) and (**c**) stability index (SI).

**Figure 3:**
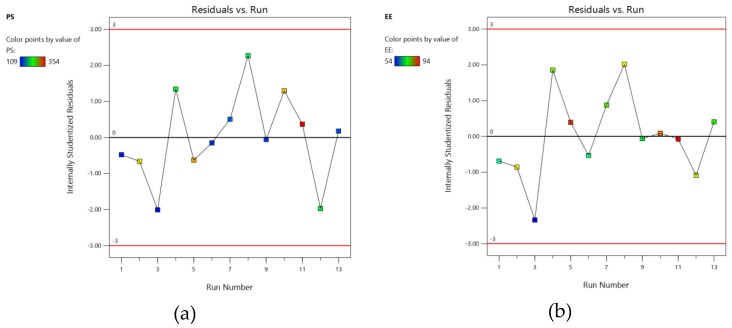

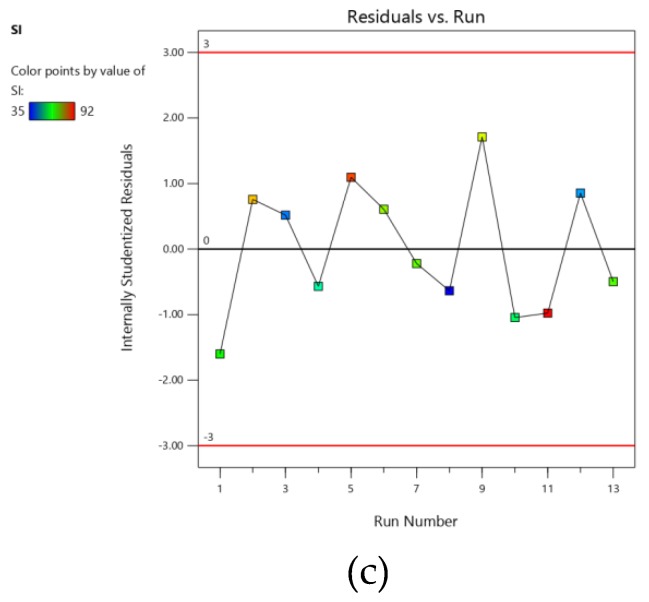
Model residuals versus test orders for (**a**) PS (**b**) EE and (**c**) SI.

A quadratic model was generated for all the responses based on the sequential sum of squares (Type I), model summary statistics and R^2^ values [24]. All the models for both responses showed a good fit with experimental data, which can be further confirmed by the high values of the adjusted coefficient of determination (R^2^). The accuracy of the model was evaluated by quantifying the derived models by studentized residuals in standard deviation units. The normal probability of the studentized residuals for the selected responses is shown in Figure 2 [25]. Moreover, Figure 3 demonstrates model residuals versus test orders to study the influence of the test orders on the derived CCD [26]. An analysis of variance (ANOVA) was performed for the inference of the quantitative effects of the factors. Data were subjected to multiple regression to yield polynomial equations. 

**Figure 4:**
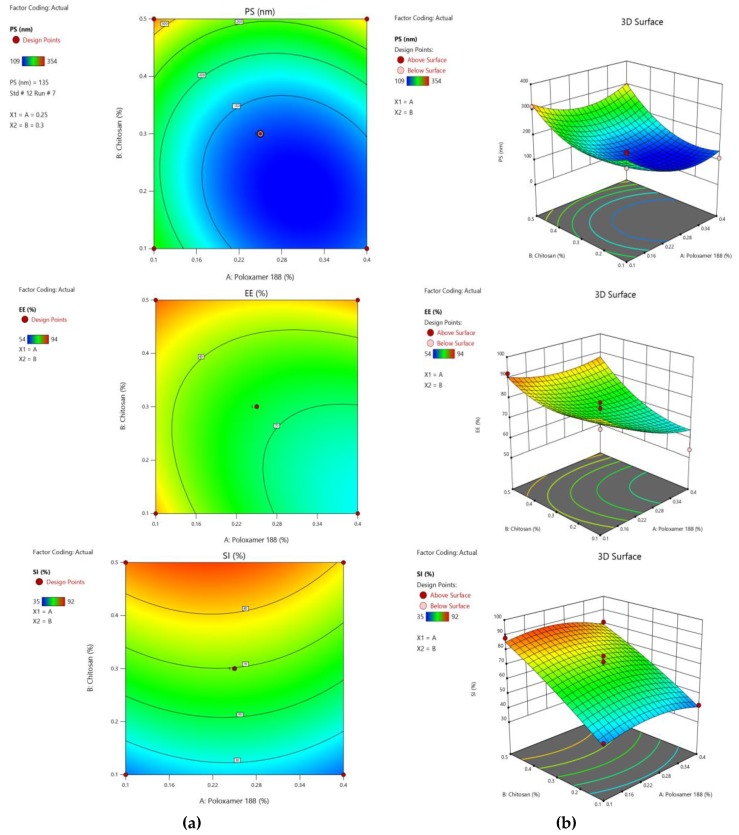
Contour plots (**a**) and three-dimensional response surface plots (**b**) for PS, EE, and SI.

Calculated F values, p values and estimated effects for the PS, EE, and SI were depicted in Table 2. These values were used to measure the significance of the coefficients of the model. The effect of independent variables on responses was further elucidated and analyzed by response surface methodology (RSM) [27]. In RSM, the main effects and interaction effects can be understood by utilizing three-dimensional response surface graphs (RSG). Contour plots give a visual representation of measured responses. Figure 4 contains a contour plot and three-dimensional response surface plots of the interaction between selected responses and the variables, which confirms the effect of the variables. The derived equations of the responses for the best fit model are given as follows:

PS = 124.20 − 31.00 A + 61.43 B + 21.25 AB + 59.03 A^2^ + 68.78 B^2^;

EE = +72.40 − 6.27 A + 6.77 B + 5.00 AB + 3.68 A^2^ + 6.18 B^2^

SI = +69.80 − 1.83 A + 20.45 B − 1.25 AB − 5.90 A^2^ − 2.15 B^2^

The optimization of different series of models obtained from the experimental analysis was done by using the global desirability function (D) [28]. All the independent variables were incorporated in the optimization of their design space. The desirability plot for the responses shows a maximum D value of 0.9154, which was obtained at optimum concentrations of independent variables. Based on this desirability approach, a formulation containing 0.24% of poloxamer 188 and 0.43% of chitosan can fulfill the prerequisites of the optimum formulation, yielding enhanced activity when loaded in ISG.

### 2.3. Preparation of ISG Loaded with SIM–QRC NPs

SIM–QRC NP-loaded ISG was prepared by using Poloxamer 407 and Carbopol 934 P. The prepared ISG was evaluated for gelation temperature, viscosity (before and after gelation), in vitro drug release, in vitro cell viability assay, and caspase-3 enzyme assay. Gelation temperature was found to be 34.1 ± at 0.4 °C. Viscosity before gelation was found to be 1.57 ± 0.68 Cp and after gelation viscosity was found to be 15.19 ± 0.70 Cp. 

### 2.4. Characterization of SIM–QRC NP-Loaded ISG Formulations

**Figure 5:**
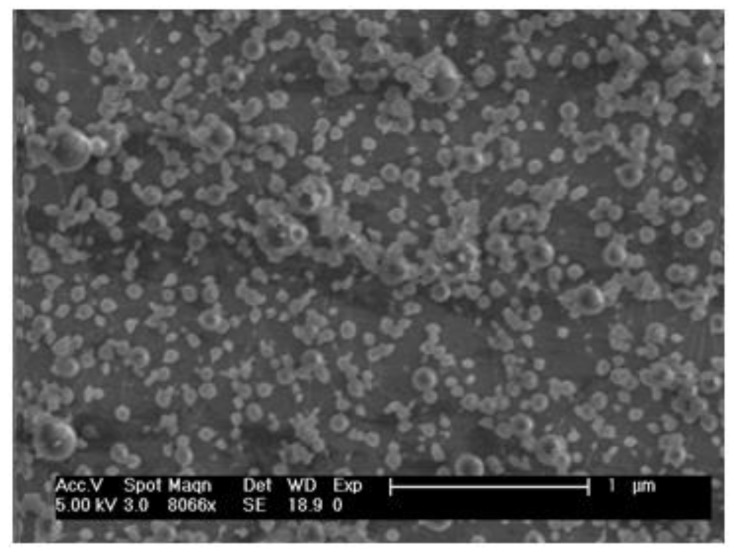
SEM image of SIM–QRC NP-loaded ISG.

SIM–QRC NP-loaded ISG was characterized for vesicle shape using a Scanning Electron Microscope (SEM) JEOL, JSM-6100, Chiyoda, Tokyo, Japan). The surface morphology of the formulations was found to be almost spherical with loose aggregates (Figure 5). The vesicle’s size distribution and zeta potential were determined by using a dynamic light-scattering process. Vesicle size was maintained uniformly and zeta potential (Zetasizer, Malvern Master Sizer 2000, Worcestershire WR14 1XZ, UK) for the optimum formulation was found to be 194 nm and 34.48 mV, correspondingly. 

### 2.5. In Vitro Release Studies

**Figure 6:**
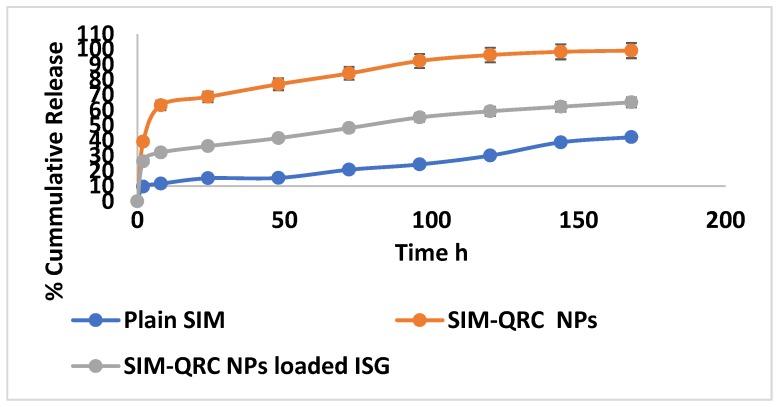
Comparative in vitro release profiles of plain SIM, SIM–QRC NPs and SIM–QRC NP-loaded in situ gel (ISG) formulations.

SIM release from the SIM–QRC NP-loaded ISG was determined by using a membraneless dissolution model and results are shown in Figure 6. Studies were carried out for up to 168 h. The cumulative percentage drug release from plain SIM, SIM–QRC NPs, and SIM–QRC NP-loaded ISG were studied as a function of time. SIM release was incomplete from plain SIM batches due to solubility issues and the release from NPs and ISG was found to be biphasic.

### 2.6. Stability Studies

Stability studies were performed by storing the samples at 4 ± 3 °C and 25 ± 2 °C, as per Q1A (R2) of the International Council for Harmonization (ICH) guidelines. Particle size and residual drug content were determined at frequent intervals of 15 days and 30 days. The results are depicted in Table 3. Under refrigeration conditions, there was not much of a change in the particle size and residual drug content, even after 30 days. At 25 ± 2 °C, there is a slight change in the particle size (at 0 day- 194 ± 3.4 nm and at after 30 days- 189 ± 3.2 nm). Residual drug content decreased to 99.0 ± 0.45% from 99.4 ± 0.28%.

### 2.7. In Vitro Cell Viability Assay

**Figure 7:**
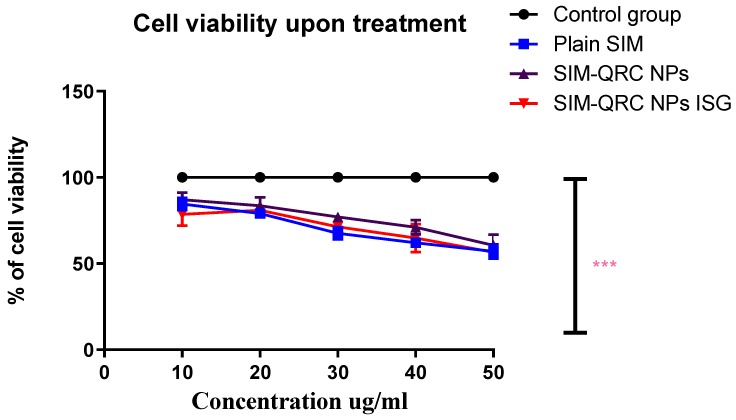
Effect of plain SIM, SIM–QRC NPs and SIM–QRC NP-loaded ISG, and 5 Fluoro-Uracil (control) on the viability of HSC-3 cell lines. The values represent the mean ± SD of three independent experiments (*n* = 9). Significantly different, (*p* < 0.05) compared to plain.

The MTT method was used to evaluate in vitro cytotoxicity against HCS-3 cells for the optimized formulation of SIM–QRC NP-loaded ISG, SIM–QRC NPs, and plain SIM after 72 h treatment. Cell viability was normalized to 100 for the control group (5 Fluoro-Uracil) and Figure 7 shows the comparison of percentage cell viability of the prepared formulations and plain SIM. This study confirms that all the treatments reduced cell viability in a dose-dependent way and SIM–QRC NP-loaded ISG showed lower cell viability than the other treatments. 

### 2.8. Caspase-3 Enzyme Assay

**Figure 8:**
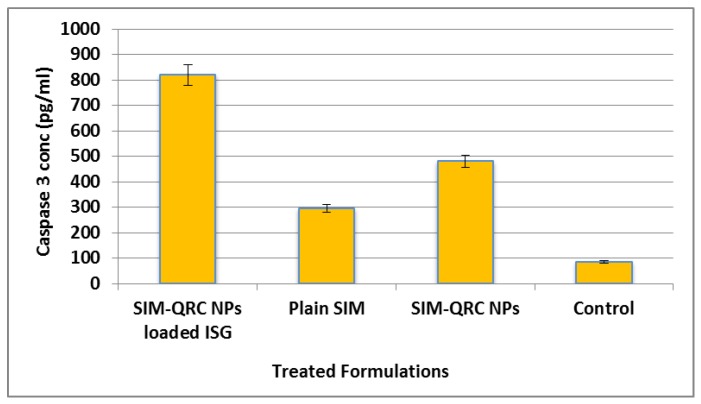
Caspase-3 enzyme concentrations in HCS-3 cells treated with different formulations, and control (solvent-free); (*n* = 6 ± SD).

The caspase-3 enzyme assay was performed by inoculating the samples in 96-well plates. A significant amount of caspase-3 was detected in HCS-3 in all the treatment formulations, as depicted in Figure 8. Caspase-3 enzyme concentration was found to be at the maximum in SIM–QRC NP-loaded ISG and minimum in the control group. 

## 3. Discussion

The characteristic peaks of pure SIM were free O-H stretch, methyl C-H symmetric and methylene C-H asymmetric stretch at 3545 cm^−1^ and 2929 cm^−1^, respectively, and lactone C-O and ester C-O stretch at 1694 cm^−1^. Methylene C-H symmetric bend and methyl C-H symmetric bend was observed at 1467 cm^−1^. The formulation spectra also show chitosan bands appearing at 3336 cm^−1^ for OH groups. All the characteristic peaks of SIM were found even in the respective formulation spectra and it confirms the compatibility between SIM and selected excipients.

The cause–effect correlations between selected parameters and the responses are described by the proposed quadratic model and their statistical significance was evaluated by ANOVA. All the selected models for PS, EE, and SI showed a good fit with the experimental data, which can be further confirmed by high R^2^ values (0.9578 for PS; 0.9157 for EE, 0.9694 for SI). Furthermore, ’Adequate precision’ is frequently used to assess signal to noise ratio (predicted response related to its associated error) and this ratio of greater than four is usually desirable for navigating design space [29]. PS, EE and SI showed a signal to noise ratio of 13.85, 16.02 and 21.02, respectively, indicating the high adequacy of the selected model. 

The obtained experimental data showed a high correlation with predicted data when presenting both the results graphically for both investigated responses. Studentized residuals were distributed along a straight line with a slight deviation (Figure 1), confirming that the modeling results were statistically acceptable [25]. Figure 2 demonstrates that residuals were scattered randomly around the centerline, but without much of a noticeable trend and they were considered independently of each other. In general, a coefficient of variation (CV) value of less than 10% normally signifies the reproducibility of the generated quadratic model. Relatively lower CV values recorded from the study (3.76% PS, 9.1% EE and 6.25% SI) confirm the reliability and accuracy of the model. If there is an inefficiency in the model in representing the data, this can be measured from the lack of fit value. The model F-value of 31.75 for PS 33.60 for EE and 44.28 for SI implies that the model is significant and there is only a 0.01% chance that a model F-value could arise due to noise. 

A nonsignificant lack of fit is required, which confirms that the model equation was appropriate in predicting the responses of any interaction. The *p* values for lack of fit were found be 0.0871, 0.0657 and 0.6677 for PS, EE, and SI, respectively. As it needs to be, lack of fit is nonsignificant [30]. The statistical significance of the quadratic equation was recognized by considering the ANOVA results. P-values less than 0.0500 indicate that the model terms are significant; values greater than 0.1000 indicate that the model terms are not significant. 

The particle size was significantly affected by (i) the antagonistic effect of A (*p*-value 0.0079), (ii) the synergistic effect of B and the polynomial terms of A, B (with *p*-values of 0.0002, 0.0003 and 0.0001, respectively), with B showing the highest effect. Response Y_2_ (EE) was significantly influenced by (i) the synergistic effect of B (with a probability value of 0.0315) and (ii) the antagonistic effect of A, with *p*-values of 0.0421 and 0.0258, respectively. Among all the significant variables, B’s effect on EE showed more magnitude. SI was affected significantly by (i) the synergistic effect of B (*p*-value < 0.0001) and (ii) the antagonistic effect of the polynomial term A, with factor B having the highest effect on SI. The effect of independent variables on responses was further elucidated and analyzed by response surface methodology (RSM). In RSM, the main effects and interaction effects can be learned by utilizing three-dimensional response surface graphs (RSG). Contour plots give a visual representation of measured responses. 

Results in Table 1 showed that the poloxamer 188 and chitosan concentrations have a large impact on the formation of NPs. When their concentrations are very low, formed nanoparticles show larger particle sizes and even higher concentrations of these factors, resulting in the formation of larger nanoparticles. This might be because of the repulsion of amino groups in chitosan molecules at higher concentrations and the addition of poloxamer 188 might promote further aggregation, which leads to precipitation, forming NPs with larger particle sizes [31]. The size of NPs can affect their distribution, for which lower liver uptake can be observed with smaller particle sizes of 25–50 nm in comparison to 150–300 nm-sized NPs and thus bioavailability. The nanosize of particles can favour penetration of cell membranes, stabilization, the binding of proteins and lyosomal escape. However, the highest EE was observed with the larger particle size and EE decreased as poloxamer 188 concentration increased. SI was even affected by chitosan concentration. Formulations with lesser chitosan concentrations were found to be unstable at room temperature. 

The optimization of the independent variables was done by setting goals for each response, then creating an overlay graph. The global desirability function (D) was applied simultaneously to optimize the series of models obtained from experimental statistical analysis. All three independent variables were incorporated in the optimization of their design space. For concurrent optimization, each response had a low and high value assigned to each goal. The PS response was set to a minimum goal, while EE and SI were adjusted to maximum goals. The desirability plot for both the responses shows a maximum D value of 0.9154, which was obtained at optimum concentrations of independent variables. Accordingly, the use of such conditions will attain a small PS (194.79 nm) and maximum EE (79.67%) and SI (82.32%). Optimized NP formulation was used to prepare ISG. 

Viscosity is one of the prerequisites for ISG. As indicated, the formulation should have a low viscosity at the time of administration and should have a high viscosity after gelation to retain it at the application site. The viscosity of SIM–QRC NP-loaded ISG measured 1.57 ± 0.68 Cp before gelation and 15.19 ± 0.70 Cp after gelation. This can be mainly because of the higher gelling of Poloxamer 407 along with carbopol, which, in turn, benefits and also retards the drug’s release for a longer period. 

Surface morphology confirms that prepared vesicles were distributed uniformly without any aggregation. The optimized formulation showed a polydispersity index (PDI) of 0.50 ± 0.04 nm, which confirms the monodispersity of the formulation. A higher zeta potential (+32.42 mV) ensured the high stability of the prepared formulation [32].

The drug release from SIM–QRC NPs and ISG is a rather complicated process and it might be affected by different factors like polymer degradation, the binding affinity of drugs with the polymer and molecular weight. SIM release from both NPs and ISG was found to be biphasic (quick release in the initial stages followed by sustained release over an extended period). At 2 h, an initial burst release was observed from SIM–QRC NPs, but at the end of 8 h, 39.23% of the drug was released in contrast with plain SIM (9.68%) and SIM–QRC NP-loaded ISG (26.30%). This initial burst release of SIM from NPs was mainly because of SIM’s presence at the surface of NPs, which allowed greater diffusion of water through the liquid matrix, which was responsible for the faster drug release. At the end of 24 h, about 68.68% of the drug was released from SIM–QRC NPs, which was reduced to 36.34% for SIM–QRC NP-loaded ISG. The drug release from plain SIM was very low because of solubility issues. Drug release from SIM–QRC NP-loaded ISG was slower to plateau up to 48.20%, but the release continued even after 96 h. However, a 99.15% cumulative drug release from SIM–QRC NPs was seen by the end of 168 h, in distinction to SIM–QRC NP-loaded ISG, which had a steady-state release of 65.12% and had the capability to continue to control the drug release beyond 168 h. This was mainly due to the gelling action of P 407 and Carbopol, which were responsible for the controlled release of the drug. The release of SIM from the other formulations was concentration-dependent and it can take place in numerous pathways such as via diffusion through the polymer matrix or through microchannels that exist in the matrix, but the drug release from NP-loaded ISG was larger because of the diffusion of SIM through the water channels of the gel matrix. 

Stability studies were performed, and percentage drug content was measured by considering an initial drug content of 100%. The measurements showed that 0.06%–1.8% of the SIM was lost from the formulation at the end of 30 days when preserved at 4.0 ± 1 °C and around 2.7% of the drug was lost from the formulations when stored at 25 ± 2 °C. For PS, there was no significant change when stored at 4.0 ± 1 °C, but there was a slight change when stored at 25 ± 2 °C. From these results, we can confirm the better stability of the formulation under refrigerated conditions, as evidenced by the higher zeta potential of the formulation. 

Three treatments (plain SIM, SIM–QRC NPs, SIM–QRC NP-loaded ISG) at different concentrations (10–50 ug/mL) were studied for cell viability along with the control group. This study confirms that all the treatments reduced cell viability with the dose (in a dose-dependent way). The observed SIM–QRC NP-loaded ISG, was lower than that of plain SIM and SIM–QRC NPs, indicating that the effect yielded higher cytotoxicity against HSC-3 cells. QRC is an antioxidant bioflavonoid and it can also significantly inhibit cell growth in a time–dose-dependent manner [33]. QRC can induce apoptosis through activation of the caspase 3-dependent pathway. Both caspase-3 enzyme assay and cell viability studies confirm that the occurrence of apoptosis with SIM–QRC NP-loaded ISG increased considerably in comparison to other treatments, this might be because we used QRC in the formulation. 

## 4. Materials and Methods

### 4.1. Materials

SIM was kindly gifted to us by Biocon Pvt. Ltd. QRC and poloxamer 188 were procured from Acros organics, Morris Plains, New Jersey, USA. Poloxamer 407, Carbopol 934 P and chitosan were purchased from Sigma-Aldrich, St. Louis, MO, USA. The National Centre for Cell Sciences (NCCS), India, gifted the HCS-3 cell lines topus. All additional chemicals, reagents, and solvents used were of analytical grade. 

### 4.2. Methods

#### 4.2.1. Attenuated Total Reflectance (ATR) Infrared (IR) Spectroscopy 

Samples for ATR-IR were prepared as per quantitative adsorption experiments. About 0.5 g of each sample was mixed with a small amount of water, then smeared directly on to the ZnSe prism crystal and analyzed in the range of 4000–650 cm^−1^. The ATR-IR spectra were collected using Nicolet’s OMNIC software in a Nicolet Nexus FTIR (Fourier-transform infrared spectroscopy) (Nicolet iS50, Thermo Scientific, Waltham, MA, USA) [34].

#### 4.2.2. Preparation of Chitosan-Coated SIM–QRC NPs 

SIM-loaded quercetin (QRC) nanoparticles (SIM–QRC NPs) were prepared by using a modified nanoprecipitation method [21]. A total of 60 mg of QRC and 5 mg of SIM were added to 12 mL of acetone. The prepared solution was added to 24 mL of aqueous phase (pH 5) containing different concentrations of poloxamer 188 and 1% acetic acid under continuous magnetic stirring. Acetone was removed by evaporation under reduced pressure until the final volume reached 10 mL. The formed nanoparticle suspensions were filtered through 1.2 µm pore size filter papers (Sartorius Stedim Biotech, Gottingen, Germany) using a vacuum pump. Chitosan-coated SIM–QRC NPs were prepared by dispersing the aqueous phase of the formulation at different concentrations of chitosan (Table 1). 

#### 4.2.3. Optimization of SIM–QRC NPs

The preparation method of chitosan-coated SIM–QRC NPs was statistically optimized by using response surface methodology (RSM) and this strategy helps in identifying the significant variables, best process conditions and to study the interaction between key variables and responses with fewer experiments [35]. Two independent variables, namely poloxamer 188 (*X*_1_) and chitosan concentrations (*X*_2_) at five different levels, plus and minus alpha (+1.414 and −1.414: axial points), plus and minus 1 (factorial points) and the center point were optimized for particle size (PS-*Y*_1_), entrapment efficacy (EE-*Y*_2_) and stability index (SI-*Y*_3_). In this experiment, face-centered central composite design (FCCCD) was employed using Design-Expert 12 software (Stat Ease Inc., Minneapolis, MN, USA) to generate 13 experimental runs. The full experimental plans in terms of actual and coded levels of parameters are represented in Table 4. ANOVA was used to establish the statistical validation of the polynomial equations generated. All the responses observed were concurrently fitted to different models. The best fitting experimental model (main, interaction and quadratic) was taken statistically based on a comparison of several statistical parameters like coefficient of variation (CV), multiple correlation coefficient (R^2^), adjusted R^2^ (Adju.R^2^) and predicted R^2^ (Pred.R^2^) [36]. The level of significance was considered at *p*-values < 0.05. A multiple factorial regression analysis (2FI for particle size and a quadratic model for EE) was carried out to measure the response (*Y_i_*) in each trial.

(1) $$Y_{i\left(2FI\right)}=b_{0}+b_{1}X_{1}+b_{2}X_{2}+b_{3}X_{3}+b_{4}X_{1}X_{2}+b_{5}X_{1}X_{3}+b_{6}X_{2}X_{3}$$

(2) $$Y_{i\left(Quadratic\right)}=b_{0}+b_{1}X_{1}+b_{2}X_{2}+b_{3}X_{3}+b_{4}X_{1}X_{2}+b_{5}X_{1}X_{3}+b_{6}X_{2}X_{3}+b_{7}X_{1}^{2}+b_{8}X_{2}^{2}+b_{9}X_{3}^{2}$$

where *Y_i_* is the dependent variable, *b*_0_ is the arithmetic mean response of all trials, *b_i_* is the estimated coefficient for factors, *X*_1_, *X*_2_, and *X*_3_ (the main effects), which are the average values of the changing factors one at a time. *X*_1_*X*_2_ and *X*_1_*X*_3_ and *X*_2_*X*_3_ represent the interaction terms and $X_{1}^{2}$, $X_{2}^{2}$ and $X_{3}^{2}$ represent the polynomial terms.

#### 4.2.4. Characterization 

##### Determination of PS and Polydispersity Index (PDI)

The PS and PDI of SIM–QRC NPs were determined by Zetasizer (Malvern Instruments, Worcestershire WR14 1XZ, UK) using a dynamic light scattering technique [37]. Samples were prepared by diluting a suitable quantity of formulation with deionized water (10 mL). Measurement conditions were as follows: temperature 25 °C, refractive index-1.33, He-Ne Red laser, 4.0 mW at 633 nm, and each measurement was performed in triplicate.

##### EE

EE was expressed in terms of the percentage of the total amount of drugs found in the prepared formulations and this was done via an indirect method. Prepared SIM–QRC NPs were taken and freeze-dried (EBT 12 N, Esquire Biotech, Chennai, India) in a petri dish and, to this dried sample, the required amount of acetonitrile was added and mixed thoroughly [38]. This was centrifuged (REMI centrifuge, C-24 BL, Chennai, India) at 10,000 RPM for about 1 h and the supernatant liquid was collected. Further washings were done with acetonitrile and all the washing contents were collected. All the washing contents were mixed with previously collected supernatant and then dried on the water bath. To this dried extract, methanol was added and diluted. Absorbance was measured at 230 nm for SIM. EE was calculated based on the following formula:

(3) $$EE\left(\%\right)=\frac{C_{total}-C_{free}}{C_{total}}$$

where *C_total_* is the theoretical amount. *C_free_* is the amount of drug detected in the supernatant SI. 

Stability studies were performed for the optimum formulation for future commercial practicability. The formulation was poured into an amber-colored glass bottle with screw cap and preserved at 4.0 ± 1 °C; 25 ± 2 °C (ICH Q1A (R2) guidelines for a period of 30 days in a stability chamber (CHM-6S, Remi Electro-Tech Ltd., Chennai, India)) [39]. At specific intervals of time, samples were withdrawn and evaluated for PS and drug content. 

#### 4.2.5. Preparation of In Situ Gel Loaded with SIM–QRC NPs

In a required quantity of deionized cold water, Poloxamer 407 (7%) and methylparaben were added and solubilized. An appropriate quantity (1 g) of prepared SIM–QRC NPs was added to the above polymer solution. Stirring was continued until the uniform solution was obtained (RQ-127 A, Remi Motor, Chennai, India). The formed solution was gelated by adding a 0.2% w/v of Carbopol 934 P under continuous stirring by using a mechanical stirrer (Remi Electro-Tech Ltd, India) at 500–1000 RPM. The final preparation was kept under refrigeration at 5 °C for about 10–12 h. This helps in the complete dissolution of polymers in the solution. Finally, the pH of the solution was adjusted by adding a small amount of triethanolamine (TEA) (LI 120, Elico, Hyderabad, India).

#### 4.2.6. Determination of Gelation Temperature

A modified Miller and Donovan method [40] was used to assess the gelation temperature of ISG. Test tubes containing around 2 mL of aliquots of the sample (in situ gel loaded with SIM–QRC NPs) were sealed with parafilm and then immersed in a water bath, maintained at a temperature of 4 °C. Then, the bath temperature was increased slowly at increments of 1 °C and left for 15 min to equilibrate at every new setting. The samples were examined for gelation temperature, where the meniscus was static on a 90° slant [41].

#### 4.2.7. Determination of Viscosity

Viscosity was measured before and after the gelation of the ISG formulation. The optimized ISG formulation was taken in a beaker and measured the viscosity by using a Brookfield viscometer (LVDV III U, Brookfield Engineering Labs, Middleborough, MA, USA) after the dipping of spindle no. 62, rotating at a speed of 50 RPM [42,43]. 

#### 4.2.8. In Vitro Release Studies

Drug release from the SIM–QRC NP-loaded ISG was determined by using a membraneless dissolution model. A total of 1 g of prepared formulation (in the cold state) was placed in a glass tube and put in a water bath of 37 °C. After the gelation of the formulation, 500 µL of pre-equilibrated phosphate buffer (pH 7.2) at 37 °C was added. At predetermined intervals of time, the complete medium was aliquoted and analyzed at λ max 230 nm in triplicate by the formerly reported high-performance liquid chromatography (HPLC) method [44]. After the sampling, the medium was replaced with an equal amount of fresh medium to maintain sink conditions.

#### 4.2.9. In Vitro Cell Viability Assay

In vitro cytotoxicity of optimized formulations of SIM–QRC NP-loaded ISG, SIM–QRC NPs and plain SIM were evaluated against HCS-3 cells using the 3-[4,5-dimethylthiazol-2-yl]-2,5-diphenyltetrazolium bromide (MTT) method. HCS-3 cells of density 5 × 10^4^ cells/mL were trypsinized using 0.25% trypsin-EDTA and added to a 96-well plate (approximately 2500-5000 cells/well). After 1 day, the total medium was replaced with Dulbecco’s Modified Eagle Medium (DMEM). Control cells were treated with 5-fluorouracil and remaining cells are treated with plain test samples at a concentration of 10 to 50 μg/mL. After incubation for about 72 h, 0.1 mL of DMEM having 0.2 mg/mL MTT was added and incubated for 2–3 h. DMEM was replaced with DMSO to dissolve the formed formazan. Then, the absorbance was measured by using a microplate reader (Biotek Synergy, Winooski, VT, USA) at 540 nm. At last, the dose–response curve was used to determine the IC _50_ (The half maximal inhibitory concentration) values [45]. 

#### 4.2.10. Caspase-3 Enzyme Assay

The caspase-3 enzyme assay was performed for SIM–QRC NP-loaded ISG, SIM–QRC NPs, and plain SIM. Initially, the cells were cultured in RPMI (Roswell Park Memorial Institute Medium) 1640 (containing 10% fetal bovine serum at 37 °C) and cell extraction buffer was used for further lysis. The collected lysate was diluted with standard buffer as per the assay range for human active caspase-3 traces and the cells were plated in DMEM (100 μL) at 1.3–1.9 × 10,000 cells/well. Each sample was inoculated in a 96-well plate 24 h before the caspase assay [46]. The assay was carried out (as per the kit instructions, USCN Life Science Inc., Wuhan, China) by using spectrophotometry at 450 nm.

## 5. Conclusions

In the present study, the poorly soluble drug SIM was productively loaded into chitosan NPs and a subsequent ISG preparation. The RSM-based FCCCD combined with the overall desirability was successfully used in optimizing the SIM–QRC NPs preparation. Based on the desirability approach, a formulation containing Poloxamer 188 0.24% and chitosan 0.43% can fulfill the prerequisites of optimum formulation for preparing SIM–QRC NP-loaded ISG. A PDI of 0.050 ± 0.04 nm confirms the monodispersity of the formulation. The formulations indicated better stability under refrigerated conditions. Higher concentrations of SIM may cause rising statin-related adverse effects such as inflammatory myopathies and rhabdomyolysis. The controlled release of SIM from SIM–QRC NP-loaded ISG can be expected to induce or can favor the angiogenesis process. ISG formulation showed a considerable increase in apoptosis occurrence through caspase-3-mediation and it also enhances the tumor suppressor protein levels. All these features make SIM–QRC NP-loaded ISG a novel formulation in creating a new channel to treat carcinomas. 

## Figures and Tables

**Table 1:** Experimental runs for face-centered central composite design (FCCCD) and observed responses.

	Factor 1	Factor 2	Response 1	Response 2	Response 3
Run	A: Poloxamer 188 (%)	B: Chitosan (%)	PS (nm)	EE (%)	SI (%)
1	0.25	0.3	114	68	64
2	0.4	0.5	294	84	81
3	0.4	0.1	109	54	42
4	0.462	0.3	218	79	54
5	0.1	0.5	314	92	88
6	0.25	0.3	121	69	72
7	0.25	0.3	135	78	69
8	0.25	0.017	208	84	35
9	0.25	0.3	123	72	76
10	0.037	0.3	305	89	58
11	0.25	0.582	354	94	92
12	0.1	0.1	214	82	44
13	0.25	0.3	128	75	68

**Table 2:** Estimated effects for the different factors in preparing SIM–QRC NPs.

Factors	PS		EE		SI	
	F-Value	*p*-Value	F-Value	*p*-Value	F-Value	*p*-Value
Model	31.75	0.0001 *	4.33	0.0408 *	44.28	<0.0001 *
A-Poloxamer 188	13.52	0.0079 *	6.16	0.0421 *	1.64	0.2417
B-Chitosan	53.07	0.0002 *	7.18	0.0315 *	203.83	<0.0001 *
AB	3.17	0.1180	1.96	0.2042	0.3807	0.5567
A²	42.60	0.0003 *	1.84	0.2168	14.75	0.0064 *
B²	57.83	0.0001 *	5.20	0.0566	1.96	0.2044
**Lack of fit**	**20.18**	0.0871 **	**5.55**	0.0657 **	**0.5629**	0.6677 **

* Significant factors. ** Nonsignificant lack of fit.

**Table 3:** Results of stability studies for SIM–QRC NP-loaded ISG at different storage conditions.

Storage Conditions	Particle Size (nm)			Residual Drug Content (%)		
	0 Day	15 Days	30 Days	0 Day	15 Days	30 Days
**4.0 ± 1 °C**	194 ± 4.7	191 ± 3.1	191 ± 4.8	99.4 ± 0.41	98.6 ± 0.38	98.9 ± 0.28
**25 ± 2 °C**	194 ± 3.4	193 ± 2.8	189 ± 3.2	99.4 ± 0.28	99.6 ± 0.27	99.0 ± 0.45

**Table 4:** Actual and coded values of formulation parameters for FCCCD design.

Independent Variables	Code and Actual Levels					Dependent Variables	Constraints
	−1.141	−1	0	+1	+1.141		
Concentration of Poloxamer 188 (%w/v)	0.037	0.1	0.25	0.4	0.462	Particle Size	Minimum
Concentration of Chitosan (%w/v)	0.017	0.1	0.3	0.5	0.582	EE	Maximum
						Stability Index	Maximum
